# Prevalence and Impact of the Relative Age Effect on Competition Performance in Swimming: A Systematic Review

**DOI:** 10.3390/ijerph182010561

**Published:** 2021-10-09

**Authors:** Jorge Lorenzo-Calvo, Alfonso de la Rubia, Daniel Mon-López, Monica Hontoria-Galán, Moises Marquina, Santiago Veiga

**Affiliations:** Departamento de Deportes, Facultad de Ciencias de la Actividad Física y del Deporte—INEF, Universidad Politécnica de Madrid, C/Martín Fierro, 728040 Madrid, Spain; daniel.mon@upm.es (D.M.-L.); monica.hontoria@upm.es (M.H.-G.); marquinascience@gmail.com (M.M.); santiago.veiga@upm.es (S.V.)

**Keywords:** young athletes, competition performance, sport talent, sport success, athlete development, talent identification, individual sport

## Abstract

This systematic review aimed to examine the prevalence of the relative age effect (RAE) in swimming and its impact on competition performance according to different types of interacting constraints. A systematic literature search, following the PRISMA guidelines for preparing systematic reviews, was performed through four electronic databases, and nine studies met the inclusion criteria. The quality of the selected studies was evaluated using STROBE, and an average score of 16.2 points was obtained. In these studies, the prevalence of the RAE in swimming was observed in more than half (58.65%) of the participants analysed, and the effect of the RAE was more accentuated in young categories (decreased as age increased and was inverted in older ones) and in male swimmers (double that in female swimmers).The impact of the RAE on competitive performance appeared to be related to the strength demands of the event, as the performance in simultaneous strokes, in shorter events, and of swimmers in the postadolescence period seems to be more affected by the RAE. These results indicate that the RAE in competitive swimming relies on individual and environmental (the swimmer’s age group and gender) but also task (the competitive events) determinants or limitations. This should serve as a guide for a more effective design of selection and development procedures for young athletes.

## 1. Introduction

In the sports field, relative age can be defined as the chronological age difference that occurs between athletes of the same age group [1]. The collection of consequences derived from this difference in birthdate is commonly referred to as the relative age effect (RAE) [2]. The RAE stipulates that those born later in the year, taking into account the cut-off date of the group (usually 1 January in swimming competitions), have a disadvantage due to later biological development [3]. This phenomenon has been generally studied in young athletes, determining a greater prevalence of the RAE in early development ages [4], as well as the possible involvement it may have in the remaining areas of their lives: education, personal relationships, and so forth [5,6].

Clustering players by competitive level, according to chronological age and based on a certain cut-off date, could lead to increasing differences affecting athletes’ performance [7,8]. Therefore, it would be logical to think that athletes born in the first months of the selection year will be over-represented, with a greater chance of reaching the highest professional sport levels. Indeed, relatively older athletes obtain higher levels of sporting success in the short term, while the long-term trend is balanced and even reversed [9]. However, the relationship between the RAE and competition performance is not clear since there are many modulating factors [10].

A lower maturational level is one of the main explanations with regard to the influence of the RAE on sport talent identification and development (TID) programs [11]. Professionals responsible for these processes (i.e., coaches, staff, stakeholders, and practitioners), with the aim of obtaining an immediate performance, tend to select athletes based on physical, anthropometric, or physiological patterns [12]. This is known as the ‘selection–maturation hypothesis’ [13]. Consequently, other types of characteristics (e.g., skill level), equally necessary for achieving high sport performance, would be obviated [14]. Furthermore, according to the constraint-based theoretical model [15], the influence of other factors on the RAE, connected to individual characteristics, such as gender or age group; sport context, such as competition category or competitive level; and/or task, that is, type of sport and event, has been highlighted. A higher prevalence in men than in women, a decrease in impact as age and/or competition category increases, and heterogeneous results in relationships between national/international competitions and individual/collective sports were observed in relation to RAEs [16,17]. This causal framework would reflect the breadth and complexity of the impact of RAEs in sport [18].

The first studies on the RAE in the sports field were carried out in team sports, such as football [19,20,21] or ice hockey [2,22]. In individual sports, the body of scientific literature is even smaller, and results suggest that RAEs are less consistent in individual sports than in team sports [23]. Even though performance requirements are more controlled, factors such as weight categories, mastery of motor skills and techniques, and competition level (for example, Olympic sports vs. non-Olympic sports) would explain the lower prevalence of the RAE in individual disciplines [11,24,25]. Swimming is a sport in which the greatest prevalence of the RAE has been observed, with a high perceived precocity in elite competition [26]. This aspect may be related to an early maturational development of swimmers and a high level of physical demand in all events of this sport [27]. Specifically, there appeared to be a large disproportion in birthdates among elite junior swimmers in England, with an over-representation of swimmers born in the first quartile of the year [27]. In addition, differences were found according to competition category and gender [28]. For example, the RAE in US elite swimmers was more prominent in males than in females [29]. However, there were no clear previous selection biases in the youth categories that affect performance; therefore, being born earlier would not be an initial advantage when competing in individual sports, except in those events in which sport performance is based more on strength, speed, and power, and there is no division into categories according to certain physical attributes, such as weight [26,30].

Swimming is a sport discipline in which muscle strength and body size have an important role in competition performance [31]. Moving through the aquatic environment requires greater energy expenditure [32], given that water is approximately 800 times denser than air, and therefore, the swimmer will have to overcome the resistance it offers in order to move forward [33]. Furthermore, only part of the force applied by the swimmer is translated into propulsive force [34], which is what allows the swimmer to move forward. Therefore, another important factor that determines success in this sport is related to the athlete’s ability to maximise propulsive force and reduce resistance to progress offered by the aquatic environment [35], which in turn is closely related to, among other aspects, the technical ability of the individual.

The different active manifestations of strength will condition the swimmer’s performance at certain moments of the event, especially the development of maximum strength [36] and explosive strength [37]. Both will determine, to a large extent, the magnitude of the traction force that the swimmer develops when swimming, as well as the jump that he/she will perform at the start or the turn push-off [38]. On the other hand, in terms of physiological variables, maximum oxygen consumption is considered an important factor that influences performance in swimming [39]. The longer the distance of the event, the greater the importance of aerobic endurance capacity [40], in addition to the role played by aerobic metabolism in short-duration events [41]. The different determinants of swimming performance according to the event are reflected on the different anthropometric characteristics described for elite swimmers, according to competitive level, specialty, and/or distance event [42]. For example, great levels of muscular endurance and low active drag forces would be required for athletes in middle-distance or distance events compared with sprinters. All these factors suggest that a great prevalence of the RAE could be expected in competitive swimming, but the impact of the RAE on performance could be dependent on the event type (i.e., duration, stroke) as this, indirectly, would have implications on the strength demands and the propulsive forces applied.

The aim of this systematic review was to conduct a systematic review to determine the prevalence of the RAE in swimming and its impact on competition performance according to the different types of interacting constraints. The scientific information published until June 2021 has been reviewed according to: (a) the individual constraints—sample characteristics (gender and age group); (b) the environmental constraints—sport context (competition category and competition level); (c) the task constraints—type of event (style and distance); and (d) the performance measurement indicators employed (type of result in competition and performance production period).

## 2. Materials and Methods

The stages of the review procedure and subsequent analysis of the original articles stayed within the guidelines set out in the Preferred Reporting Items for Systematic Reviews and Meta-Analyses (PRISMA) checklist and the Population, Intervention, Comparison, Outcomes, and Study Design (PICOS) question model for the definition of the inclusion criteria [43,44].

### 2.1. Search Strategy

Primary and original studies for the purpose of evaluating the association between the RAE and competition performance in swimming were included. Furthermore, studies had to have been published in any language in peer-reviewed journals with an impact factor included in the Journal Citation Reports of the Web of Science (JCR of WoS) or Scimago Journal and Country Rank (SJR of Scopus) until June 2021.

The Preferred Reporting Items for Systematic Reviews and Meta-Analyses (PRISMA) statement was followed for the literature review. A search of the scientific literature was conducted using Web of Science (WoS), PubMed, Sport Discus, and Google Scholar as databases. The predefined search strategy was carried out using terms grouped into three search strings: (1) ‘RAE’ or ‘relative age’ or ‘relative age effect*’ or ‘birthdate effect*’ or ‘age effect*’ or ‘influence of age’; and (2) ‘swimming’ or ‘swimming event*’ or ‘front crawl’ or ‘freestyle’ or ‘breaststroke’ or ‘backstroke’ or ‘butterfly’ or ‘medley’ or ‘aquatic sport*’ or ‘professional sport*’ or ‘elite sport*’ or ‘youth sport*’; and (3) ‘performance’ or ‘ranking’ or ‘classification’ or ‘place’ or ‘medal*’ or ‘success’ or ‘attainment’ or ‘selection’ or ‘competitions times’ or ‘stroke distance’.

### 2.2. Eligibility Criteria

According to the ‘PICOS’ question model, the inclusion criteria were: (1) population: swimmers over the age of 10 years old with the highest standard of performance who participate in the first or second competition levels (international or national swimming competitions) or the third competition level (competitions involved in talent identification and development systems) [45]; (2) intervention: national and international official high-performance competitions with official statistics about competition performance; (3) comparison: association between competition performance and swimmer’s birthdate in relation to individual, environmental, and task constraints; (4) outcomes: prevalence and impact of the RAE on competition performance according to one specific indicator, “performance period” (short term and/or long-term), and based on gender, age group, competition category, competition level, and type of event (style and distance); and (5) study design: observational–descriptive research based on an the impact of the RAE on competition performance in swimming.

**Figure 1:**
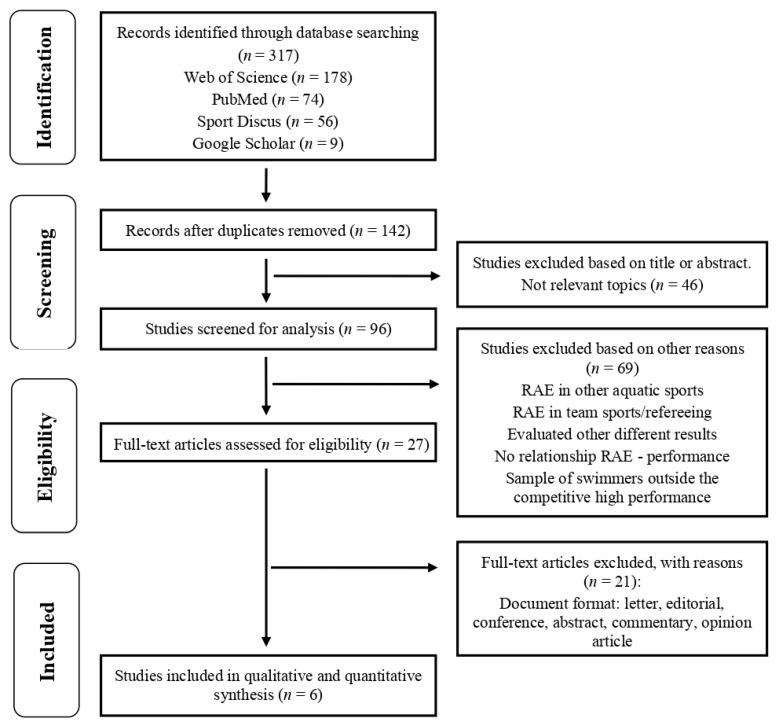
Flow chart for screening and selection according to the Preferred Reporting Items for Systematic Reviews and Meta-Analyses (PRISMA).

The authors worked separately and independently to ensure the reliability of the process and the suitable eligibility of the studies. According to the criteria for preparing systematic reviews, PRISMA, the protocol was carried out in the months of May and June 2021, and it was made up of four stages (Figure 1): (1) identification: the first (J.L.-C.) and second authors (A.d.l.R.) found 317 studies in the four digital databases; and (2) screening: the first author (J.L.-C.) eliminated the duplicate files (*n* = 142) and excluded those considered not relevant through a previous reading of the title, abstract, and keywords (*n* = 96). Furthermore, the first author (J.L.-C), jointly with the second (A.d.l.R.) and third (M.M.), rejected studies linked to instability according to the exclusion criteria through a full-text reading (*n* = 58): (3) eligibility: the first (J.L.-C.), second (A.d.l.R.), and fourth authors (S.V.) eliminated full-text studies from the selection process by the eligibility criteria (*n* = 27); and (4) inclusion: the remaining studies (*n* = 6) based on the relationship between the RAE and swimming performance were finally considered.

### 2.3. Study Selection and Data Extraction

A standardised form was used to extract data from the studies included in the review for assessment study quality and scientific evidence. In order to conduct an in-depth analysis, the sample of the studies under review was distributed into different subcategories according to a constraints-based theoretical model [15]. Furthermore, the samples were classified according to the presence or lack of the RAE and, within those with the RAE, according to the influence on competition performance. Data associated with the samples (‘*n*’) and swimmers (‘*n*’ and ‘%’) were provided.

- Individual Constraints:
  Based on the sample characteristics (A), the swimmers were grouped according to: (A1) gender: male and female; and (A2) age group: preadolescent (10–12 years), adolescence (13–14 years), postadolescence (15–18 years), and adults (>18 years) [16,27].
- Environmental Constraints:
  In relation to the sport context (B), the swimmers were allocated according to: (B1) competition level: national or international; and (B2) competition category: U-11, U-12, U-13, U-14, U-15, U-16, U-17, U-18, and senior.
  According to the grouping method (C), the swimmers were categorised based on the birthdates and official cut-off dates approved by the corresponding national or international federation. Thus, the swimmers were divided into annual competition cycles by quartile: Quartile 1 (Q1), swimmers born between 1 January and 31 March; Quartile 2 (Q2), swimmers born between April 1 and June 30; Quartile 3 (Q3), swimmers born between July 1 and September 30; and Quartile 4 (Q4), swimmers born between October 1 and December 31.
- Task Constraints:
  With regard to the type of event (D), the swimmers were allocated according to: (D1) style: front crawl/freestyle, breaststroke, backstroke, butterfly, and medley; and (D2) distance: short distance (50 and 100 m), medium distance (200 and 400 m), and long distance (800 and 1500 m).
- Sample Distribution:
  In terms of sample distribution (E), the set of swimmers in each of the studies was categorised according to the composition of the sample based on birthdate: RAE (heterogeneous distribution of birthdates in an annual competition cycle) or no RAE (homogeneous distribution of birthdates in an annual competition cycle).
- Relationship between the RAE and Competition Performance:
  The samples were grouped based on the influence of the RAE on competition performance according to performance period (G). Thus, the swimmers were included in one of the following groups: (G1) impact/no impact of the RAE on short-term competition performance (statistical parameters associated with short competitions such as competition times, medals, and points); (G3) impact/no impact of the RAE on long-term competition performance (attainments throughout the sport career and maintenance periods in talent systems or rankings).

### 2.4. Study Quality Assessment

To determine the quality of the studies, a modified version of the ‘Strengthening the Reporting of Observational Studies in Epidemiology (STROBE)’ checklist [46] was used. This checklist was composed of 22 items clustered into six categories belonging to the different sections of the study: ‘Title–Abstract’ (item 1), ‘Introduction’ (items 2–3), ‘Methods’ (items 4–12), ‘Results’ (items 13–17), ‘Discussion’ (items 18–21), and ‘Funding’ (item 22). A score of ‘−’ was assigned to incomplete items, and ‘+’ to items that were described accurately. The overall rating was obtained from the summation of the item values based on the following levels: ‘very low quality’ (0–4 points), ‘low quality’ (5–8 points), ‘medium quality’ (9–12 points), ‘high quality’ (13–16 points), and ‘very high quality’ (17–22 points). The quality assessment of the studies was carried out by two independent reviewers (J.L.-C. and A.d.l.R.). Another reviewer (M.M.) resolved disagreements in the rating, and inter-rater reliability was calculated.

## 3. Results

### 3.1. Study Characteristics

Of the six studies analysed, four studies included a mixed sample in terms of gender [47,48,49,50], while the other two were composed of only men [51] or women [52]. All the studies were focused on formative categories (11–18 years) and national competitions, except the one carried out by Ferreira et al. [48], which was found to be made up of a senior sample that participated in an international competition (i.e., Olympic Games). The six included studies used the annual competition cycle as the sample grouping method, categorising swimmers into birth quartiles (Q1–Q4). Four of the six studies focused on analysing more than one style [47,48,49,50], while two of them did so on a single style, either breaststroke [52] or front crawl [51]. Finally, competition performance in four studies was expressed as competition times or FINA points [47,50,51,52], while only one of them did so through medals [48], and another one through selection and reselection processes [49]. A more comprehensive description of the samples and swimmers included in these studies in this systematic review can be found in Table 1 and Table 2. The studies are arranged chronologically to favour the interpretation and longitudinal interpretation and evaluation of the findings.

### 3.2. Prevalence of the RAE

The prevalence of the RAE based on the sample characteristics (IC), sport context, (EC) and type of event (TC) is shown in Table 3. Of the six studies included in this systematic review, 294 independent samples and 42,466 swimmers were identified, although differences were detected depending on the sample composition. The sample distribution was heterogeneous in 121 samples (*n* = 24,906 swimmers, 58.65%) with the presence of the RAE, while in 173 samples without the RAE (*n* = 17,560 swimmers, 41.35%), the distribution was homogeneous. Therefore, the prevalence of the RAEs was higher in terms of the number of swimmers but lower in terms of the number of samples.

Considering the sample characteristics–individual constraints (A), the following results were found: (A1) Gender: The prevalence of the RAE was higher in male than in female swimmers (ratio, 2.1:1 vs. 1:1). In female competitions, even more samples were notably identified with no presence of the RAE (*n* = 95). (A2) Age group: The magnitude of the RAE in formative sport categories decreased as the chronological age of the swimmers increased (preadolescence, ratio, 11.7:1; adolescence, ratio, 1.2:1; and postadolescence, ratio, 0.3:1). Therefore, the RAE was dissolving throughout the sport transition process. In the adult development stage, only two samples with a balanced RAE/no RAE (ratio, 0.9:1) were examined.

With regard to the sport context–environmental constraints (B), the following was observed: (B1) Competition category: The evolutionary trend of the RAE is similar to the behaviour of the ‘age group’ variable. The most accentuated prevalence of the RAE took place in the U-12 category (*n* = 5464 swimmers; 12.87%), while the highest number of swimmers with no presence of the RAE was found in the U-15 and U-18 categories (*n* = 4028; *n* = 3.816, respectively). Interestingly, all the samples analysed in the U-11 category (*n* = 9; 3414 swimmers) were affected by the RAE. (B2) Competition level: In national competitions, a greater number of swimmers affected by RAEs were detected (*n* = 24,434) in comparison with those with lack of the RAE (*n* = 17,053) at a ratio of 1.4:1. However, the number of samples biased by birthdate was lower (*n* = 120) in comparison with those not biased (*n* = 172). At the international level, only two samples with a balanced RAE/no RAE ratio (0.9:1) were examined.

In relation to the type of event–task constraints (D), the following data were observed: (D1) Style: The styles with the highest prevalence of the RAE were the butterfly (ratio, RAE/no RAE, 1.5:1) and the medley (ratio, RAE/no RAE, 1.8:1). Conversely, more cases with no presence of the RAE were identified in the backstroke style (ratio, 1.8:1). (D2) Distance: The ratio between the RAE/no RAE was reduced as the event distance increased (short events, ratio, 1.5:1; medium events, ratio, 1.4:1), and the relationship was reversed in long-distance events (ratio 0.4:1).

### 3.3. Impact of the RAE on Competition Performance

The impact of the RAE on competition performance based on the sample characteristics and sport context is shown in Table 4. Among the samples (*n* = 121) and swimmers (*n* = 24,906) who were biased by birthdate, more cases with an influence of the RAE on swimmers’ competition performance were observed (*n* = 22,361; 89.78%). According to the performance period, a greater impact of the RAE on short-term competition performance was identified, both in the number of samples (*n* = 75) and in the number of swimmers (*n* = 22,126). Conversely, the impact of the RAE on long-term competition performance was only detected in 2 samples composed of 235 swimmers.

**Figure 2:**
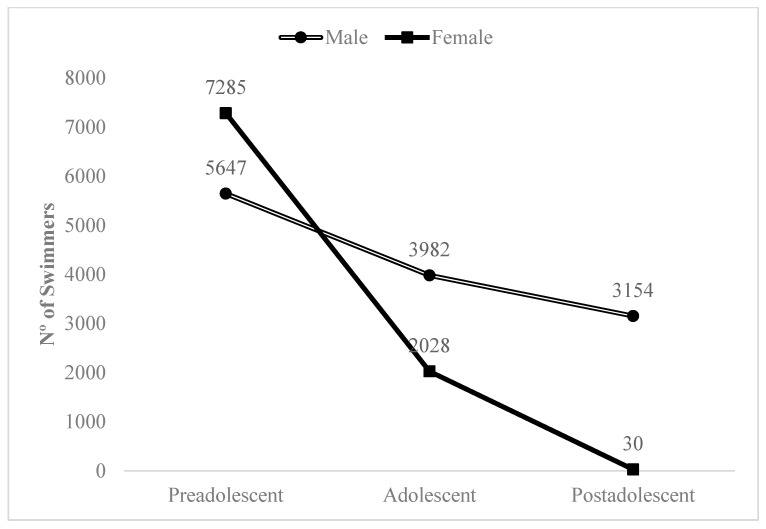
Impact of the RAE on competition performance according to gender and age group.

In relation to the sample characteristics–individual constraints (A), the following results were found: (A1) Gender: The RAE affected short-term competition performance to a greater extent in male swimmers (ratio, impact/no impact, 11.8:1) than in female swimmers (ratio, impact/no impact, 6.4:1). In the long term, only 2 samples of male and female swimmers showed an influence of the RAE on competition performance. (A2-B1) Age group/competition category: The impact of the RAE on short-term competition performance did not decrease progressively as the age of the swimmers increased. Thus, in the postadolescent stage (U-16/U-18) the impact/no impact ratio was 12.5:1, while lower ratios in the preadolescent (U-11/U-13) and adolescent (U-14/U-15) stages were detected (11.5:1; 8.7:1, respectively). However, if the cases with an impact of the RAE on performance were observed exclusively by combining gender and age group, a downward evolutionary trend was identified as the swimmer’s age increased and was more notable in women than in men (Figure 2). In the adult stage (senior), only 1 sample was identified with no impact of the RAE on short-term competition performance.

With regard to the sport context–environmental constraint (B), the following was observed: (B2) Competitive level: In national competitions, a predominant impact of the RAE on short-term competition performance was observed in 88.84% of the swimmers (impact/no impact ratio, 10.7:1). However, 45 samples with no impact of the RAE were registered (*n* = 2074 swimmers). At the international level, only 1 sample (*n* = 471 swimmers) with no impact of the RAE on short-term competition performance was identified.

**Figure 3:**
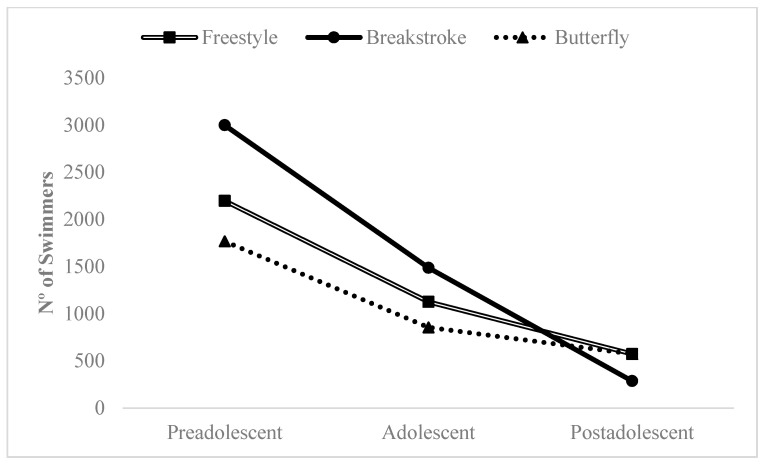
Impact of the RAE on competition performance in short-distance events according to age group and style.

According to the type of event–task constraints (D), the following data were observed: (D1) Style: The greatest impact of the RAE on short-term competition performance was observed in breaststroke events (impact/no impact ratio, 33.2:1). In the other styles, a notable impact of the RAE was also detected (butterfly, ratio, 14.1:1; medley, ratio, 10.6:1; and freestyle, ratio, 8.3:1), except in the backstroke style, in which more samples (*n* = 9) and swimmers (*n* = 405) with no influence of the RAE on competition performance were found. (D2) Distance: The impact of the RAE on short-term competition performance decreased as the event distance increased (short-distance events, ratio, 18.3:1; and medium-distance events, ratio, 8.3:1). Combining the event types with the highest impact of the RAE (freestyle, breaststroke, and butterfly in short distances) with the age group, a decrease in the influence of the RAE on competition performance was also observed as the swimmer’s age increased, the most notable being in breaststroke (Figure 3). In long-distance events, only 4 samples (*n* = 184 swimmers) with no influence of the RAE on competition performance were identified.

### 3.4. Study Selection and Assessment (Quality Analysis)

The quality analysis (‘RAE–Performance Strengthening the Reporting of Observational Studies in Epidemiology (STROBE)’ checklist) yielded the following results (Table 5): (a) The quality scores ranged from 14 to 19. (b) The average score was 16 points. (c) Of the six included studies, four (66.67%) were categorised as ‘high quality’ (13–16 points), and two (33.33%) were considered ‘very high quality’ (17–22 points). The highest scores were located in the ‘Introduction’ (100%), ‘Discussion’ (87.5%), ‘Results’ (75%), and ‘Methods’ (70.37%) sections. The lowest scores were in the ‘Funding’ section (33.33%). Among the highest-quality studies, we considered that item no. 2 (background/rationale), no. 3 (objectives—‘state specific objectives and/or any prespecified hypothesis’), no. 4 (study design), no. 5 (setting), no. 6 (participants), no. 11 (descriptive results—‘the number (absolute frequency) or percentage (relative frequency) of participants found in each grouping category and subcategory’), no. 12 (statistical methods), no. 13 (participants), no. 14 (descriptive data), no. 16 (main results), no. 18 (key results—‘a summary of key results concerning study objectives’), and no. 21 (generalisability) were considered complete (100%). By contrast, the most commonly absent or incomplete items (0 points) were no. 7 (variables), no. 15 (outcome data), no. 17 (other analysis), and no. 22 (funding).

## 4. Discussion

The aims of the present systematic review were to examine the prevalence and impact of the RAE in competitive swimming according to the different types of interacting constraints. Differences in the chronological age distribution between athletes of the same age group were observed in 58.65% of swimmers analysed. From all of them, an impact on competition performance was detected in 89.78% of the sample. The RAE was more present in younger ages, male swimmers, and some specific events, the impact on performance being more notable in the short term. Despite the prevalence of the RAE in swimming having been previously reported, this is the first study to analyse and detect, according to the different constraints, an impact of the RAE on swimming competition performance.

### 4.1. Prevalence of the RAE according to Constraints-Based Model

The greater presence of athletes born in Q1 compared with the rest of the quartiles (see Table 1) was in line with previous data in individual sport disciplines [16,53] and team sports [17], although a great variability of results was observed between studies. Variation in birthdate within the same age group is a key factor that must be taken into account as athletes of the same category may be trained in the same group but with very different stages of development between them [54]. Elite adolescent swimmers who are born in the first quarter of the year are more likely to present greater height or body mass index (BMI) than their counterparts [54], and consequently, they could perform better in swimming competitions. Indeed, swimmers who reach the elite level usually present some physical characteristics, such as greater development in body height and ecto-mesomorphic development [55]. Specifically, BMI has been associated with swimming performance and fitness level in young athletes [56]. Furthermore, relatively older swimmers, due to a greater maturational status [57], are also expected to present greater muscle growth [27] as arm, leg, and back muscular strengths are important determinants of swimming performance [31,55]. The complex interaction of all these anthropometric in addition to neuromuscular (i.e., strength–power, muscle growth, and muscle architecture changes) gains during the pubertal stage could probably explain the effects of chronological age differences between athletes.

However, the prevalence of the RAE in competitive swimming is opposite to that in other aquatic disciplines, such as artistic swimming or diving, where the role of greater development in body height or strength is smaller [23]. These are predominantly technical disciplines where performance is measured through a scoring system and where some previous studies have observed a lower impact of the RAE [13] or even a reverse RAE [58]. This is known as the ‘underdog effect’, that is, a greater prevalence of swimmers born in the last quarter of the year compared with those born in the first quarter [59]. Additionally, the reverse RAE has been observed at the master’s swimming level, producing an advantage for the relatively younger athletes (in principle, with better physical characteristics) within the same age-group category [29].

In the present study, the prevalence of the RAE seemed to decline with age, and there was a clear effect between the ages of 12 and 14 years in both male and female swimmers [26,50,51,52]. After that, the influence of the RAE seemed to fade away, especially around the ages of 17–18 years, before completely disappearing in elite competition [48]. These results are in line with previous studies in swimming and in other sport disciplines, where competition begins at a young age and which suggest that the RAE is more present at lower sport levels than in more advanced ages [26,47]. The explanation for this phenomenon might be associated with age at peak height velocity (APHV) and puberty occurring in the 11- to 14-year-old period, which, undoubtedly, increases differences in physical attributes between athletes born in different quartiles [60]. Indeed, previous countries beginning elite training at an early age compared with the other continents (like in the Asian continent) have been reported to be more affected by the RAE [48]. This creates a problem in the medium and long term for the athletes themselves since, according to previous studies [20,61], a swimmer who succeeds at a younger age has a greater tendency to continue in the practice of swimming due to greater motivation. Greater success at this age can cause athletes to set more demanding long-term objectives for themselves [62] or increase their self-confidence and the perception of competition [63], in addition to greater choices for sports development (i.e., access to better training structures, higher tiers of competition, or more experienced coaches), as previously suggested [64]. On the other hand, athletes who see their chances of success increasingly frustrated by rivals who have superior characteristics to theirs can have a higher rate of dropout from the sport. For example, in the master’s category, a lower percentage of success in their category intensifies the involvement of external factors (i.e., economic, social, or family) and causes a higher rate of dropout [29]. From another point of view, there is evidence that, after the PHV occurs, late-maturing athletes present an increased progression in anthropometric and physical attributes and “catch up” the early-maturing athletes during adolescence and at adulthood [65,66]. This, undoubtedly, should be considered in the talent identification programs as the selection bias in the early stage of athlete development could be not beneficial in the long term.

Concerning gender, no previous results for the RAE has been drawn up that can be qualified as conclusive. A more pronounced RAE was observed in the male gender according to FINA performance [47], whereas women were found to be more strongly influenced [48], or no differences among genders were found [49]. What the data seem to reveal is that there is an earlier decline in the impact of the RAE on women [26] according to the difference in maturational development compared with that in men. Women bring forward their physical development by a few months, or even years, depending on the case [39], and this could influence the tendency of the RAE to fade earlier. The results of the present study indicate that the prevalence of the RAE in male swimmers was double that in female swimmers (see Table 1). This could also be linked to the anthropometric and floatability differences between genders [67], which undoubtedly reinforces the role of strength in performance in male swimmers. Thus, in individual sports such as swimming, female categories, as the competition level increases, have a more limited pool of selectable players available [16]. A high sport dropout throughout the swimmers’ formative period causes the RAE to progressively lose strength and, therefore, have less impact on competition performance.

### 4.2. Impact of the RAE on Competition Performance and Talent Identification Programs

In relation to competitive performance, the greatest impact of the RAE on swimming performance was observed in the postadolescent period (U-15/U-18 categories) and for the butterfly and 400 m medley events. This could be related to swimmers in this age group achieving the greatest strength gains, which directly influenced their competitive outcomes [68]. Indeed, simultaneous strokes (such as the butterfly or breaststroke, both present in the medley events) require greater strength levels from swimmers due to a greater centre of mass velocity variations during the stroke cycle [69]. On the other hand, alternative strokes (freestyle and backstroke) present lower velocity variations within the stroke cycle [70], which is in line with the lower impact of the RAE on competition performance. A change in competitive performances due to the RAE was observed in the present study at the national level, as not enough samples were collected for international-level events. Indeed, international competition in swimming usually begins at ages 15–16 years, where the impact of the RAE is considerably diminished. Differences in the influence of the RAE were also observed depending on the event distance, as longer-event performances seemed to be less affected by the RAE. This was in line with previous comments on the strength effect on performance, as greater strength levels are required to maximise performance on shorter compared with longer swimming events [71]. Swimmers born in the last part of the year are probably more specialised in longer events, where they can succeed through a greater volume of practice despite their theoretical lower strength and maturational status. This is clearly reported when the age of peak performance of longer versus shorter events is explored in swimming [72]. Those events where strength demands are greater present older years of peak performance.

From a practical point of view, the presence of the RAE could affect proper talent identification and selection. There is evidence that the younger the athlete and the further away they are from peak performance, the more uncertainty later international success may entail [72,73]. Indeed, the RAE showed its greatest impact in the years associated with growth and maturation, and it appeared earlier among females, as they may mature earlier [39]. Since competition times refer to the technical and physical level of an athlete, this leads to the RAE in relation to the maturation–selection hypothesis and having an influence on selection processes [20]. Athletes selected at early ages will have more chances to experiment with practice of high-standard and quality feedback from experienced coaches [74]. However, the influence of task constraints (i.e., strokes or distance) on the RAE should also be considered when designing an intervention with young developing athletes. According to the presented results, some strokes and events present a greater impact of the RAE on competition performance than others. Therefore, changes of performance in determined events could be tracked with the athlete’s growth in order to clearly distinguish the RAEs from long-term chances of success in the sport.

### 4.3. Limitations

The main limitation of the present study was that the final sample was composed of a small number of studies (*n* = 6). This may limit the generalisability and extrapolation of the findings, which should be considered with caution. Lack of data in lower levels made it impossible to select more studies. The absence of individual valuation statistics beyond the result itself could also compromise the findings. Therefore, the results must be accurately interpreted.

### 4.4. Future Research

To the best of our knowledge, this is the first systematic review conducted on the relative age effect on competition performance in swimming. Thus, some future research lines are proposed based on this study.

According to a more in-depth analysis, subsequent studies should include a meta-analysis that would provide more accurate statistical information on the behaviour of the RAE in competition performance in swimming.

A solid theoretical framework on the RAE in swimming in relation to its main demands could clarify the topic [18]. Besides, conducting longitudinal studies to see trends in swimming could help in the diagnosis of the RAE. 

On the other hand, increasing the sport levels investigated in swimming could help to make a complete framework of the impact and influence of the RAE on competitive performance and, therefore, adjust policies for identifying sport talent. For example, establish in-depth relationships on how the RAE behaves (prevalence and impact) in different types of swimming events, that is, combining style and distance.

Lastly, a greater number of cohort studies in the RAE literature may provide the most appropriate study design to capture the nuances of the RAE problem, focusing on corrective adjustment procedures that remove relative age-related participation and performance inequalities in swimming events. Thus, the participation experience of swimmers would be improved, and on the other hand, evaluation tests would be more precise [52].

## 5. Conclusions

Differences in chronological age distribution between competitive swimmers from the same age group were observed in 58.65% of the cases analysed, with increased participation in championships for those born in the first quarter. From all of them, an impact on performance was detected in 89.78% of the sample. The RAE was more present in younger ages and male swimmers, and its prevalence diminished with age, producing the reverse RAE in senior categories. The impact of the RAE on competition performance was more noticeable in the short term, and it probably did not support the hypothetical benefit of the selection bias in the early stage of athlete development. The impact of the RAE on performance seemed to be related to the event’s strength demands, as it affected, to a greater extent, the performance in simultaneous strokes, in shorter events and on swimmers in the postadolescence period. Therefore, the RAE in competitive swimming seemed to rely on individual and environmental (the swimmer’s age group and gender) but also task (the competitive events) constraints and this should serve as a guide for a more effective design of selection and development procedures for young athletes.

## Figures and Tables

**Table 1:** Distribution of the sample according to the characteristics of the swimmers, sport context, grouping method, and sample distribution based on birthdate.

Author (s)	Sample Characteristics (IC)			Sport Context (EC)		Type of Event (TC)	Grouping Method (EC)	Sample Distribution
	*n*	Age	Gender	Competition Category	Competition Level	Style (Distance (m))		
Costa et al. (2013) [47]	473	11–12	M	U-12	2010 Portuguese swimming competition events (NL)	Fc (50, 100, 200, 400, and 1500); Bc (100 and 200); Br (100 and 200); Bf (100 and 200); Md (200 and 400)	By quartile (Q1–Q4)	RAE
	650	12–13	M	U-13				RAE
	649	13–14	M	U-14				RAE
	650	14–15	M	U-15				RAE
	634	15–16	M	U-16				RAE
	586	16–17	M	U-17				No RAE
	439	17–18	M	U-18				RAE
	624	11–12	F	U-12		Fc (50, 100, 200, 400, and 800); Bc (100 and 200); Br (100 and 200); Bf (100 and 200); Md (200 and 400)		RAE
	650	12–13	F	U-13				RAE
	644	13–14	F	U-14				RAE
	623	14–15	F	U-15				No RAE
	519	15–16	F	U-16				RAE
	392	16–17	F	U-17				RAE
	280	17–18	F	U-18				RAE
Ferreira et al. (2017) [48]	507	20–28	M	Senior	London 2012 Olympic Games (IL)	All Olympic events combining style (Fc, Bc, Br, Bf, and Md) with distance (50, 100, 200, 400, 800/1500, and 10,000)	By quartile (Q1–Q4)	No RAE
	471	18–26	F	Senior				RAE
Nagy et al. (2018) [49]	118	11–12	M	U-12	Talent program—Hungarian Swimming Association (NL)	All events combining style (Fc, Bc, Br, Bf, and Md) with distance (50, 100, 200, 400, 800/1500, and 10,000)	By quartile (Q1–Q4)	RAE
	117	11–12	F	U-12				RAE
Cobley et al. (2019) [51]	488	12–13	M	U-13	Australian state/national competitions (NL)	Fc (100)	By quartile (Q1–Q4)	RAE
	548	13–14	M	U-14				RAE
	566	14–15	M	U-15				No RAE
	538	15–16	M	U-16				No RAE
Abbott et al. (2020) [52]	657	11–12	F	U-12	Australian state/national competitions (NL)	Br (100)	By quartile (Q1–Q4)	RAE
	643	12–13	F	U-13				RAE
	635	13–14	F	U-14				RAE
	556	14–15	F	U-15				No RAE
	444	11–12	F	U-12		Br (200)		RAE
	432	12–13	F	U-13				RAE
	436	13–14	F	U-14				No RAE
	386	14–15	F	U-15				No RAE
Staub et al. (2020) [50]	1724	10–11	M	U-11	German Swimming Federation (NL)	Fc (50 and 400); Br (50 and 200); Bf (100); Md (200)	By quartile (Q1–Q4)	RAE
	1724	11–12	M	U-12				RAE
	1711	12–13	M	U-13				RAE
	1711	13–14	M	U-14				RAE
	1723	14–15	M	U-15				RAE
	1722	15–16	M	U-16				RAE
	1720	16–17	M	U-17				RAE ^†^
	1718	17–18	M	U-18				No RAE ^†^
	1690	10–11	F	U-11				RAE
	1680	11–12	F	U-12				RAE
	1689	12–13	F	U-13				RAE
	1588	13–14	F	U-14				RAE^†^
	1671	14–15	F	U-15				No RAE ^†^
	1657	15–16	F	U-16				No RAE
	1684	16–17	F	U-17				No RAE
	1697	17–18	F	U-18				No RAE

Notes: *n* = absolute frequency of the sample; IC = individual constraints; EC = environmental constraints; TC = task constraints; M = male; F = female; U-11 = under 11; U-12 = under 12; U-13 = under 13; U-14 = under 14; U-15 = under 15; U-16 = under 16; U-17 = under 17; U-18 = under 18; NL = national level; IL = international level; Fc = front crawl; Bc = backstroke; Br = breaststroke; Bf = butterfly; Md = medley; Q1–Q4 = birth quarter; No RAE = no relative age effect; RAE = relative age effect; ^†^ = except in some events.

**Table 2:** Relationship between the relative age effect (RAE) and competition performance providing the aim(s) of the study, performance indicators, main results, and conclusions.

Author (s)	Aim (s) of the Study	Performance	Main Results (RAE–Performance)	Conclusions
Costa et al. (2013) [47]	To analyse the birthdate distribution in both genders and the respective mean performance points for the main individual swimming events	FINA points based on competition times	1. At a general level, relatively older swimmers obtained higher individual performance parameters than relatively young swimmers 2. Based on the gender and competition category, the RAE affected competition performance in male cadet (U-12), infantile (U-13 and U-14) and juvenile swimmers (U-16), and female junior swimmers (U-16) 3. According to the competition category and the event, the RAE had an impact on the competition performance in the 100 m butterfly event in female infantile swimmers (U-12) and in the 200 m breaststroke event in female junior swimmers (U-15). However, in the female U-15 category, the swimmers who obtained the best performance were those born in Q2	No impact of the RAE on short-term individual performance in most of the indicators analysed
Ferreira et al. (2017) [48]	This study aims at investigating the relative age effect on Olympic swimmers who participated in the London 2012 Olympic Games by analysing the possible differences among continents between sexes and verifying the relationship with winning Olympic medals	Medals	1. Relatively older swimmers did not achieve results (based on the number of successful levels of Olympic medals) significantly better than relatively young swimmers	No impact of the RAE on short-term individual performance
Nagy et al. (2018) [49]	The major aim of which was to explore the characteristics of the newly implemented talent management program and its effects on the selection and development of talented junior swimmers	Selection and reselection in the talent program (more than 1 year)	1. The swimmers born in the first 3 months of the year (Q1–Q3) are still more likely to be recruited to the Hungarian talent program than their relatively younger counterparts (Q4) 2. Furthermore, as a potential effect of the new program, the dominance of the first quarter of the year (Q1) is also characteristic among those eligible for the next level of talent management	Impact of the RAE on long-term individual performance
Cobley et al. (2019) [51]	The purpose of the present study was first to generate accurate estimates of the relationship between decimal age (i.e., chronological and relative age) and swimming performance based on longitudinal competition data	Competition times	1. Competition performance was affected by the RAE in the U-13, U-14, and U-15 categories (top 50%/20%/10% of the swimmers). However, no impact of the RAE was found on the overall number of swimmers in the U-16 category 2. The impact of the RAE on competition performance decreased as the relative age/decimal age of the swimmers increased	Impact of the RAE on short-term individual performance, except in the U-16 category
Abbott et al. (2020) [52]	The purposes of the present study were first to accurately estimate the longitudinal relationship between decimal age (i.e., chronological and relative) and performance in female breaststroke swimming	Competition times	1. Competition performance was affected by the RAE in the U-12/13/14/15 categories (top 25%/10% of the swimmers) in 100 m breaststroke events, except for the top 10% of the U-15 2. Competition performance was affected by the RAE in the U-12 and U-13 categories (top 25%/10% of the swimmers) in 200 m breaststroke events. However, no impact of the RAE was found on the overall number of swimmers in the U-14 and U-15 categories 3. The impact of the RAE on competition performance decreased as the relative age/decimal age of the swimmers increased	Impact of the RAE on short-term individual performance, except in the U-15 category
Staub et al. (2020) [50]	This investigation aims to quantify the prevalence, magnitude, and transient pattern of RAE across a German cohort of age-group swimmers according to sex and event.	Competition times	1. Competition performance was affected by the RAE in the U-11, U-12, U-13, U-14, U-15, and U-16 categories (top 100 male swimmers) in the six events analysed. With regard to the U-17 category, the impact of the RAE was detected on 50 m and 400 m freestyle, 100 m butterfly, and 200 m individual medley events. In the U-18 category, the impact of the RAE was only found in 400 m freestyle events 2. Competition performance was affected by the RAE in the U-11, U-12, and U-13 categories (top 100 female swimmers) in the six events analysed. With regard to the U-14 category, the impact of the RAE was detected in 400 m. freestyle, 50 m breaststroke, 100 m butterfly, and 200 m individual medley events. In relation to the U-15, U-16, U-17, and U-18 categories, the RAE had no impact on competition performance, except on 200 m individual medley events in the U-15 category	Impact of the RAE on short-term individual performance (depends on the event) in men (from 10 to 16–17 years) and in women (from 10 to 13–15 years) No impact of the RAE on short-term individual performance (depends on the event) in men (from 17 to 18 years) and in women (from 14–15 to 18 years)

Notes: FINA = International Swimming Federation.

**Table 3:** Summary of the sample’s distribution (*n* and %) according to the relative age effect (RAE or no RAE) by characteristics of swimmers (gender and age group), sport context (competition category and competition level), and type of event (style and distance).

Constraint	Subgroup Category	RAE			No RAE		
		Samples	Swimmers		Samples	Swimmers	
		*n*	*n*	%	*n*	*n*	%
Sample Characteristics (IC)	**Gender**						
	Male	67	13,980	32.92	78	6619	15.59
	Female	54	10,926	25.73	95	10,941	25.76
	**Age Group**						
	Preadolescence (10–12)	68	14,291	33.65	27	1225	2.88
	Adolescence (13–14)	33	6706	15.79	49	5680	13.38
	Postadolescence (15–18)	19	3438	8.10	96	10,148	23.90
	Adult (>18)	1	471	1.11	1	507	1.19
Sport Context (EC)	**Competition Category**						
	U-11	12	3414	8.04	0	0	0
	U-12	32	5464	12.87	10	375	0.88
	U-13	24	5413	12.75	17	850	2.00
	U-14	23	4559	10.74	18	1652	3.89
	U-15	10	2147	5.05	31	4028	9.49
	U-16	10	1882	4.43	29	3188	7.51
	U-17	7	1238	2.92	31	3144	7.40
	U-18	2	318	0.74	36	3816	8.99
	Senior	1	471	1.11	1	507	1.19
	**Competition Level**						
	National	120	24,435	57.54	172	17,053	40.16
	International	1	471	1.11	1	507	1.19
Type of Event (TC)	Style *						
	Freestyle—front crawl	43	8179	19.83	63	5931	14.38
	Backstroke	10	435	1.05	18	763	1.85
	Breaststroke	29	8439	20.46	39	5998	14.54
	Butterfly	17	3425	8.30	27	2334	5.66
	Medley	19	3722	9.02	25	2027	4.91
	Distance *						
	Short distance	52	12,550	30.42	74	8618	20.89
	Medium distance	62	11,466	22.79	88	8020	19.44
	Long distance	4	184	0.45	10	415	1.01

Notes: *n* = absolute frequency; % = relative frequency; no RAE = no relative age effect; RAE = relative age effect; IC = individual constraints; EC = environmental constraints; TC = task constraints; U-11 = under 11; U-12 = under 12; U-13 = under 13; U-14 = under 14; U-15 = under 15; U-16 = under 16; U-17 = under 17; U-18 = under 18. * Due to lack of data about the type of event (style and distance) in some studies, the analysis of the sample by these subgroup categories was reduced to 41,253 swimmers.

**Table 4:** Summary of samples (*n*) and swimmers (*n* and %) with regard to the relationship between the RAE and short-term competition performance by sample characteristics (gender and age group), sport context (competition category and competition level), and type of event (style and distance).

Categories	Subgroup Categories	Impact—RAE			No Impact—RAE		
		Samples	Swimmers		Samples	Swimmers	
		*n*	*n*	%	*n*	*n*	%
Gender	Male	43	12,783	51.32	23	1079	4.34
	Female	30	9343	37.51	23	1466	5.89
Age group—competition category	Preadolescent (U-11/U-13)	42	12,932	51.93	24	1124	4.51
	Adolescent (U-14/U-15)	19	6010	24.14	14	696	2.79
	Postadolescence (U-16/U-18)	12	3184	12.78	7	254	1.02
	Adult (senior)	0	0	0	1	471	1.89
Competition level	National	73	22,126	88.84	45	2074	8.33
	International	0	0	0	1	471	1.89
Type of event (style) *	Freestyle—front crawl	24	7306	30.19	19	873	3.61
	Backstroke	1	30	0.12	9	405	1.67
	Breaststroke	24	8193	33.86	5	246	1.02
	Butterfly	12	3198	13.21	5	227	0.94
	Medley	12	3399	14.05	7	323	1.33
Type of event (distance) *	Short distance	38	11,900	49.17	14	650	2.69
	Medium distance	35	10,226	42.26	27	1240	5.12
	Long distance	0	0	0	4	184	0.76

Notes: *n* = absolute frequency; % = relative frequency; RAE = relative age effect; STIP = short-term individual performance; U-11/U-13 = under 11–13; U-14/U-15 = under 14–15; U-16/U-18 = under 16–18. * Due to lack of data about the type of event (style and distance) in some studies, the analysis of the sample by these subgroup categories was reduced to 24,200 swimmers.

**Table 5:** Study quality assessment based on the adapted version of Strengthening the Reporting of Observational Studies in Epidemiology (STROBE).

Items STROBE	Costa et al. (2013) [47]	Ferreira et al. (2017) [48]	Nagy et al. (2018) [49]	Cobley et al. (2019) [51]	Abbott et al. (2020) [52]	Staub et al. (2020) [50]
*1. Title/abstract. Informative/balanced summary of what was done and what was found is provided	+	+	+	+	+	+
*2. Background. Scientific background and rationale for the investigation being reported is explained	+	+	+	+	+	+
*3. Objectives. State specific objectives and/or any prespecified hypothesis	+	+	+	+	+	+
*4. Study design. Present key elements early	+	+	+	+	+	+
*5. Setting. Setting, locations, and relevant dates for data collection are described: study period, sport context, and competition year(s) for all data	+	+	+	+	+	+
*6. Participants. Give characteristics of the sample (overall number, age, gender)	+	+	+	+	+	+
*7. Variables. Clearly define all outcomes, exposures, predictors, potential confounders, and effect modifiers	-	-	-	-	-	-
*8. Data source. Source and procedure for obtaining the birthdate and performance sample characteristics are described	-	-	-	-	+	+
*9. Bias. Describe any efforts to address potential sources of bias	-	-	+	-	-	-
*10 Study size. Explain the study size						
*11. Statistical methods. Statistical methods, including specific analytical methods used to examine subgroups and interactions (relationship RAE–performance), are described	+	+	+	+	+	+
*12. Statistical methods. How duplicates and missing data were addressed or incomplete data were handled (if applicable) is explained	+	+	+	+	+	+
*13. Descriptive results. The number (absolute frequency) or percentage (relative frequency) of participants found in each grouping category and subcategory is reported	+	+	+	+	+	+
*14. Main results. Statistical estimate and precision (i.e., 95% IC) for each sample or subgroup is provided	+	+	+	+	+	+
*15. Main results. Post hoc comparisons (OR) between grouping categories are provided	-	-	-	-	-	-
*16. Main results. A measure of effect size is provided (i.e., Cramer’s V, phi coefficient, Cohen’s)	+	+	+	+	+	+
*17. Main results. A coefficient of correlation between RAE and performance measures is provided	-	-	-	-	-	-
*18. Key results. A summary of key results with reference to study objectives is provided	+	+	+	+	+	+
*19. Limitations. Limitations, considering sources of potential bias or imprecision, are discussed	+	+	+	-	-	+
*20. Interpretation. An overall interpretation of results considering objectives/evidence is provided	+	+	+	-	+	+
*21. Generalisability. The generalisability of the study results to similar or other contexts is provided	+	+	+	+	+	+
*22. Funding. The funding source of the study is cited, or the lack of funding, if applicable	-	-	-	-	+	+
SCORE	15	16	17	14	16	18

Notes: Title/abstract = *1; Introduction = *2–*3; Methods = *4–*12; Results = *13–*17; Discussion = *18–*21; Funding = *22; ‘0′ = item with absence or lack of information; ‘1′ = item with complete and explicit information.
